# A Life on the Frontlines: The Legacy of Norman Bethune (1890–1939)

**DOI:** 10.7759/cureus.67286

**Published:** 2024-08-20

**Authors:** Krystal Rampersad, Michael Montalbano

**Affiliations:** 1 Medical Education and Simulation, St. George’s University School of Medicine, St. George’s, GRD; 2 Anatomical Sciences, St. George’s University School of Medicine, St. George’s, GRD

**Keywords:** armed conflicts, blood transfusion services, social responsibility, surgeons and physicians, historical vignette

## Abstract

Henry Norman Bethune was a prominent Canadian thoracic surgeon who came to fame during the 1930s. After being made a Fellow of the Royal College of Surgeons of Edinburgh, Bethune became head of thoracic surgery in a hospital in Cartierville, Canada. During this time, he pioneered surgical techniques, published research findings, and invented surgical instruments. Not content with being only a physician, innovator, and humanitarian, Bethune also found himself in medical services on the frontlines of wars in both Spain and China. In Spain, Bethune emphasized the need for prompt blood transfusions and developed mobile blood transfusion services. After the start of the Second Sino-Japanese War, Bethune traveled to China and quickly organized a mobile operating unit. Following discussions with Chinese leaders, Bethune performed surgeries on the frontlines of conflict in northern China, where his exceptional loyalty to duty became famous throughout the region. Although he met his end at an early age due to septicemia in 1939, his medical legacy carries on in multiple countries and serves to inspire a future generation of medical practitioners.

## Introduction and background

Dr. Norman Bethune was a Canadian physician, an innovator in the field of medicine, and an altruist whose impact extended far beyond his home country. His early life was marked by a keen interest in science and a commitment to helping others, laying a foundation for his future career in medicine and humanitarian work. Personally affected by tuberculosis, his early career saw him working as a physician who made significant contributions to the treatment of illnesses that afflict the indigent. As a civilian doctor, Bethune would go on to be a highly productive pioneer in both surgical operations and crafting medical devices, garnering worldwide attention during his time as a head of thoracic surgery in Canada. However, Bethune is most renowned for his work on blood transfusion services during the Spanish Civil War and surgical units in the Second Sino-Japanese War, both of which demonstrated his dedication to advancing medical care even under the most challenging circumstances. While his life was tragically cut short when he died of an infected wound while operating in China, Bethune’s legacy continues to inspire medical professionals and activists around the world.

## Review

Early life and education

**Figure 1:**
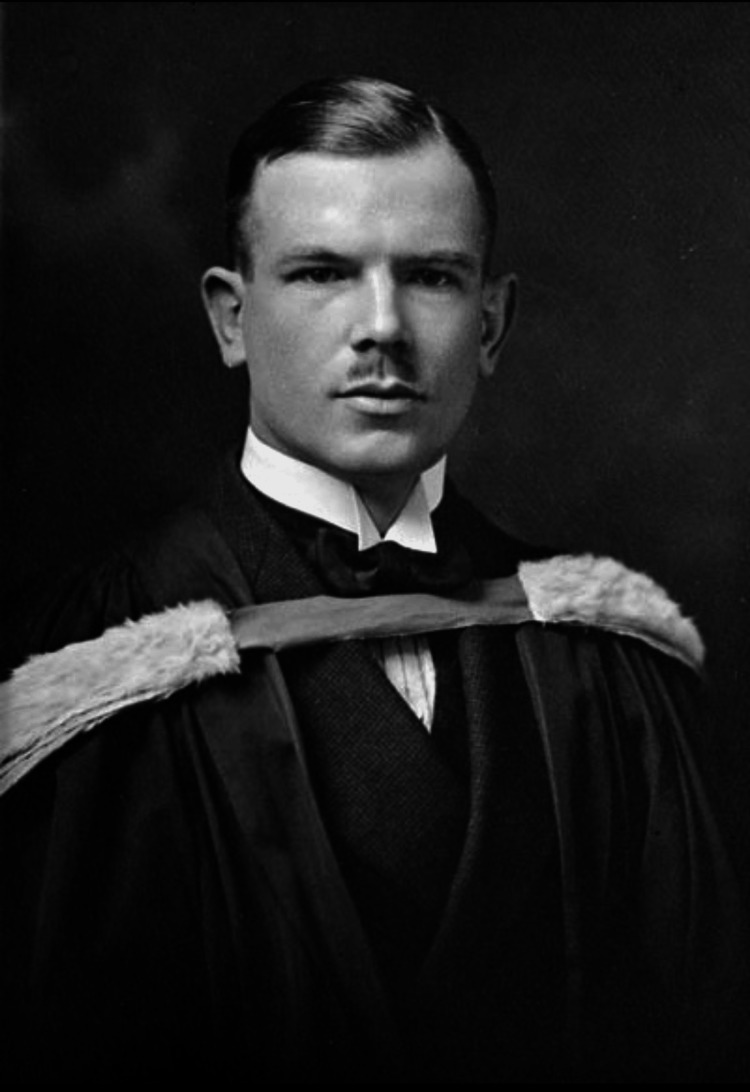
Norman Bethune, graduation photo. Image obtained from Library and Archives Canada/PA-160708 (Public Domain). Source: https://www.collectionscanada.ca/.

Henry Norman Bethune was born in Gravenhurst, Ontario in 1890. By age eight, he insisted on being called Norman in honor of his grandfather who served as a military surgeon in the Crimean War [1-3]. He entered the University of Toronto in 1909 to further his education in the field of biochemistry and physiology and then entered medical school in 1912 [4,5]. This formative medical training was interrupted due to financial difficulties that required him to leave medical school. During this time, he completed odd jobs as a lumberjack, an English teacher, and a reporter to finance his medical training. He also temporarily paused his studies to enlist with the Royal Canadian Army Medical Corps in 1914 after the entry of Canada into World War I [5,6]. While enlisted and stationed in France, Bethune suffered a leg wound that led to hemorrhagic shock and an eventual medical discharge from duty in 1915 [2]. On his return home, he completed his education and graduated from medical school (Figure 1). Afterward, he returned to duty, serving first as a lieutenant surgeon on the HMS Pegasus and then as a medical officer with the Canadian Flying Corps [5]. These experiences in the armed forces and exposure to the challenges of military medicine contributed significantly to his later innovations and overall dedication to medical care.

Initial career path

Dr. Bethune was made a Fellow of the Royal College of Surgeons of Edinburgh in 1922 after completing further studies at Edinburgh University [2,5]. Of note, this qualification was the same as that held by his grandfather, which reflects Bethune’s commitment to upholding his familial legacy [7]. In 1924, with only $24 to his name, he relocated to Detroit, Michigan. There he rented an apartment in the red-light district, which he also utilized as his private practice office, and became a voluntary assistant in the surgical outpatient department at Harper Hospital by 1925 [5]. He also took an additional part-time position as an instructor at the Detroit College of Medicine and Surgery where he conducted various classes and notably continued educational discussions with students at a local speakeasy [5].

In 1926, Norman contracted bilateral tuberculosis. At a time before the use of streptomycin and when the disease carried a case fatality rate of nearly 50%, he moved to a sanatorium for the recommended treatment of rest. However, his illness worsened, and he became bedridden until January 1927 [5]. As a patient, Bethune convinced a medical resident to perform an artificial pneumothorax treatment on his left lung after reading Dr. John Alexander’s “Surgery of Pulmonary Tuberculosis.” This led to a 65% collapse of his lung, followed by recovery and discharge a few months later after becoming sputum-negative [4,5]. While this led to a short-term clinical improvement, air would periodically reaccumulate and lead to recurrent pulmonary symptoms. To further resolve these symptoms, Bethune would self-administer pneumothoraces and eventually underwent a left phrenectomy [1,5]. This period in a sanatorium with tuberculosis would profoundly deepen Dr. Bethune’s social consciousness as he came to intimately understand the links between environmental factors such as poverty and their connection with illness and disability.

Civilian surgical career

After his convalescence, Bethune was accepted as a fellow in thoracic surgery at McGill University by a pioneer of thoracic surgery, Dr. Edwin Archibald [4,8]. However, this acceptance was contingent on Bethune completing three months under Dr. David T. Smith at Ray Brooks State Hospital in New York. Bethune would successfully complete this preliminary work, with Dr. Smith later remarking that in the three-month period, Bethune learned more about bacteriology than most graduate students did in three years. Afterward, Bethune performed nearly 300 operations in the subsequent year as a fellow. This included 60 thoracoplasty operations, 53 lipiodol injections, and 43 phrenicectomy procedures [6].

However, differences of opinion led to various challenges as Archibald believed Bethune was “quick but rough, not careful, far from neat and just a little dangerous” in his surgeries [6]. Despite their disagreements, Archibald remained convinced of Bethune’s capabilities and suitability for thoracic surgery. In 1932, Dr. Archibald was successful in having Bethune appointed head of thoracic surgery at l'Hôpital du Sacré-Cœur. However, this was after nearly a year of Dr. Archibald persuading an archbishop and other hospital authorities that a “Protestant anglophone” such as Bethune was worthy of a post in a “Roman Catholic francophone hospital” after the fellowship [5].

Medical innovations and advocacy for reform

This new position allowed Bethune to continue his pioneering work in thoracic surgery and further establish his legacy in the medical field. An innovator in the medical field, Bethune made significant contributions to thoracic surgery and medical instrumentation. In pioneering procedures, he was the first physician to perform bilateral thoracoplasties, the first Canadian to perform pneumonectomy in a patient under 10 years old, and the first to insufflate talc in the pleural space, a technique that is still used today [3,4]. He also designed many surgical instruments that have been further refined and used in modern medicine. These include devices such as periosteal scrapers, table-mounted scapula retractors, long handle rib shears, and a “phrenectomy necklace” used to hide surgical neck scars [3,5,6,8].

Throughout his career, Bethune’s devotion to self-improvement was notable. He was renowned for his self-critical approach, exemplified by his presentation “Some Errors in Technique and Mistakes in Judgment made in the course of 1000 Thoracic Surgical Operations” at the 1936 annual meeting of the American Association for Thoracic Surgery. While his subsequent paper on the same topic was rejected at the time by a journal editor for being “too provocative,” it highlights his lifelong dedication to learning and medical advancement [3].

During this period, he authored 14 papers as a thoracic surgeon such as “A Plea for Early Compression in Pulmonary Tuberculosis.” In his publications, Bethune recognized the larger impact of the environment and politics on human health, writing that “the rich man recovers, the poor man dies” [3]. In practice, this position became advocacy both for clinicians’ financial stability and the safeguarding of patient health. At the patient level, this included providing healthcare services free of charge in his clinic on Saturday mornings in Verdun [3,6]. At an institutional level, inspired by an international conference in Leningrad chaired by Ivan Pavlov, Bethune would form the Montreal Committee for Security of the People’s Health and propose medical reforms to the Medico-Chirurgical Society of Montreal [1,3]. Bethune’s proposals would be rejected, and the Medico-Chirurgical society would expel him for his ideas and “unconventional dress” [1].

Advances in battlefield medicine

Spurned by Canadian medical societies and drawn to the anti-fascist struggles in Europe, Dr. Bethune traveled to Spain in November 1936. Here, Bethune combined his knowledge of transfusion techniques involving citrated blood with the importance of prompt transfusion to develop mobile blood transfusion services in the Spanish Civil War [2]. Aided by the support of Spanish Republican radio and press efforts, the transfusion service had enrolled over 1,000 donors and collected approximately 80 units (10 gallons) by January 1937. By March of 1937, Bethune was promoted from Major to Comandante and his team was servicing up to 100 transfusions per day on a 1,000 km front [2,9]. As a result, it is estimated that a total of 1,900 transfusions were performed that same year [2]. Over the course of the war, the efforts of Bethune’s team are estimated to have reduced mortality by 30% [2].

In 1938, Bethune traveled to China following the capture of Beijing by Japanese military forces in the Second Sino-Japanese War. After landing in Hong Kong, Bethune traveled over 600 miles to the headquarters of the Chinese Eighth Route Army and joined the Red Army Medical Corps [8]. Once again advocating that physicians need to be on the frontlines providing care to the wounded, Bethune formed an integral part of the “Canadian-American Medical Unit.” This unit consisted only of himself, two Chinese physicians, an interpreter who doubled as an anesthesiologist, a cook, and two orderlies. As a precursor to the mobile army surgical hospital (MASH) units of World War II, this group functioned to carry medical supplies by mules to the frontlines of battle to tend to the wounded using a collapsible operating table designed by Bethune [6]. Bethune also trained and graduated Chinese peasants to become “barefoot doctors” within a year, wrote a manual for guerilla warfare medical practice, and worked to convert Buddhist temples into hospital facilities [1,3]. These successes would lead to his appointment as a medical advisor for Jin-Zha-Ji Military District with more than 20,000 hospital units reporting to him by 1939.

However, that same year he succumbed to septicemia secondary to a finger injury sustained in an operation to repair a fractured tibia. As Bethune’s guerilla medical techniques are estimated to have saved thousands, his death was a great loss to the Chinese. His death was followed by a heartfelt eulogy from Mao Zedong and he was buried in Revolutionary Martyr’s Cemetery in Shijiazhuang, Hebei [5,8].

Posthumous international recognition

Following his death, Bethune’s lifetime of service has been recognized across many countries. Among his medical contemporaries who volunteered in the Spanish Civil War, Dr. Leo Eloesser wrote that “None of our members [in Spain] has had more stirring actions, and few, perhaps more useful ones, crowded into the years of his life than Norman Bethune” [5]. As a result, Bethune’s name was given to Spanish streets in both Malaga and Motril [10].

In his country of birth, a documentary on Bethune was commissioned by the National Film Board of Canada in 1964. However, wider distribution ceased after Canada kowtowed to U.S. pressure that opposed Bethune’s political leanings [5]. In 1972, coinciding with expanding diplomatic and economic ties with China, the Canadian government remembered their wayward national and officially pronounced Dr. Bethune “a Canadian of national historical significance” [8]. His birthplace, a manse in Gravenhurst, became a national historic site known as Bethune Memorial House in 1976 [3]. Additionally, Canada established Norman Bethune College at York University, Ontario, and Dr. Norman Bethune Collegiate Institute secondary school in Scarborough, Ontario [8].

In China, multiple recognitions have been given in honor of Bethune, who is also known as Pai-Ch'iu-En or “white one sent” [6]. On the centenary of Bethune’s birth in 1990, China issued commemorative stamps in his honor [8]. In 1991, the Norman Bethune Medal was established as a premier medical honor in China [8]. His name also adorns multiple institutions across China, including the Norman Bethune Health Science Center of Jilin University, Bethune Military Medical College, Bethune Specialized Medical College, and Bethune International Peace Hospital [8].

## Conclusions

Dr. Henry Norman Bethune was more than a medical doctor, he valued human life to an exalted degree that demanded he put the interests and health of others before his own. Bethune’s spirit of innovation would ultimately shape his legacy as a talented doctor, a trailblazer in surgery, and a pioneer in the use of blood transfusions on the frontlines of battlefields. Forever a relentless fighter against societal inequities, Bethune’s profound commitment to healing and humanitarianism made him a generous benefactor to the underprivileged across the United States, Canada, Spain, and China. As a stalwart humanitarian, his legacy as a selfless and courageous physician lives on in the hearts of the patients he treated and the lives he inspires with the possibilities of medicine.
